# Personalized prescription of tyrosine kinase inhibitors in unresectable metastatic cholangiocarcinoma

**DOI:** 10.1186/s40164-018-0113-x

**Published:** 2018-09-06

**Authors:** Elena V. Poddubskaya, Madina P. Baranova, Daria O. Allina, Philipp Y. Smirnov, Eugene A. Albert, Alexey P. Kirilchev, Alexey A. Aleshin, Marina I. Sekacheva, Maria V. Suntsova

**Affiliations:** 1 I.M. Sechenov First Moscow State Medical University (Sechenov University), Moscow, 119991 Russia; 2 Clinical Center Vitamed, 10, Seslavinskaya St., Moscow, 121309 Russia; 3 Pathology Department, Morozov Children’s City Hospital, 4th Dobryninsky Lane 1/9, Moscow, 119049 Russia; 4 State Research Center‐Burnasyan Federal Medical Biophysical Center of Federal Medical Biological Agency, Moscow, 123098 Russia; 5 Stanford University School of Medicine, Stanford, CA 94305 USA; 6 D. Rogachev Federal Research Center of Pediatric Hematology, Oncology and Immunology, Moscow, 117198 Russia

## Abstract

**Background:**

Cholangiocarcinoma is an aggressive tumor with poor prognosis. Most of the cases are not available for surgery at the stage of the diagnosis and the best clinical practice chemotherapy results in about 12-month median survival. Several tyrosine kinase inhibitors (TKIs) are currently under investigation as an alternative treatment option for cholangiocarcinoma. Thus, the report of personalized selection of effective inhibitor and case outcome are of clinical interest.

**Case presentation:**

Here we report a case of aggressive metastatic cholangiocarcinoma (MCC) in 72-year-old man, sequentially treated with two targeted chemotherapies. Initially disease quickly progressed during best clinical practice care (gemcitabine in combination with cisplatin or capecitabine), which was accompanied by significant decrease of life quality. Monotherapy with TKI sorafenib was prescribed to the patient, which resulted in stabilization of tumor growth and elimination of pain. The choice of the inhibitor was made based on high-throughput screening of gene expression in the patient’s tumor biopsy, utilized by Oncobox platform to build a personalized rating of potentially effective target therapies. However, time to progression after start of sorafenib administration did not exceed 6 months and the regimen was changed to monotherapy with Pazopanib, another TKI predicted to be effective for this patient according to the same molecular test. It resulted in disease progression according to RECIST with simultaneous elimination of sorafenib side effects such as rash and hand-foot syndrome. After 2 years from the diagnosis of MCC the patient was alive and physically active, which is substantially longer than median survival for standard therapy.

**Conclusion:**

This case evidences that sequential personalized prescription of different TKIs may show promising efficacy in terms of survival and quality of life in MCC.

**Electronic supplementary material:**

The online version of this article (10.1186/s40164-018-0113-x) contains supplementary material, which is available to authorized users.

## Background

Cholangiocarcinoma (CCA) is a bile duct cancer that is mainly characterized by its late diagnosis and fatal outcome [1]. CCA accounts about 3% of all gastrointestinal tumors and is second most common liver tumor after hepatocellular carcinoma [2]. Overall 5-year survival rate is lower than 10% [3], while overall 1-year survival of patients at stage 4 is only 5% [4]. Treatment options for CCA include surgery and chemotherapy, but only about 30% of patients are available for surgery [5].

Standard care chemotherapy treatment for CCA is Gemcitabine, which is administered alone or in combination with cytotoxic agents such as Cisplatin. The response rate ranges from 8 to 60%, depending on the cohort of patients [6]. Nevertheless, these patients have a poor prognosis with a median survival of 6–12 months [7]. Thus, there is a need for improvement of general CCA treatment options.

Currently there are several ongoing clinical trials utilizing different approaches for target therapy in CCA. Chimeric antigen receptor-modified T (CART) cell-based therapy was recently attempted in CCA [8]. Authors reported 8.5- and 4.5-month partial response to CART-EGFR and CART-CD133 therapy respectively. Nevertheless, several associated side effects were discovered, from which epidermal damage was the most prominent. Indeed, so far CART showed limited efficiency in solid tumor and further studies are still required for successful clinical applications [9].

Majority of clinical trials investigate the efficacy of small molecule inhibitors acting at different levels of EGFR signaling pathway, which is known for being upregulated in CCA (summarized in the recent review [10]). Several case reports [11–13] on TKI usage in CCA demonstrated potential benefits from such treatment. However, results of clinical trials so far are controversial. Luo et al. in non-controlled and single arm study showed that Sorafenib in combination with best clinical practice had modest effect for the patients with advanced CCA [14]. At the same time, Sorafenib monotherapy had been shown to be beneficial in a cohort of 15 patients [15], but larger studies demonstrated that Sorafenib as a single agent had rather low activity in cholangiocarcinoma [16, 17]. In turn, Pazopanib in combination with another TKI drug Trametinib showed a trend to increase 4-month progression-free survival as compared with the prespecified null hypothesized 4-month PFS of 25%. However, this trend did not reach statistical significance [18]. Previously reported treatment outcomes described above are summarized in Table 1. Differential efficiency of TKIs between studies and individual patients within a study could be explained by the range of factors from genetic heterogeneity [19] to patient ethnicity [20]. This illustrates that despite the potential benefits for a distinct cohort of CCA patients there are difficulties associated with the correct TKI prescriptions.

**Table 1:** Results of published case reports and clinical trials for TKI usage in CCA

Drug	Number of patients	Dose	Duration of treatment	Outcome	Side effects	Refs.
Sorafenib	2	400 mg PO bid	4 months with later switch to oxaliplatin and gemcitabine; 6 month for the time of report	Transient disease stabilization; decrease of tumor markers CA 125, CA 19-9, CA 27.29	Maculopapular rash, hair thinning, grade 3 thrombocytopenia (disappeared after 1-week discontinuation), hypertension, facial rush	[13]
Sorafenib	1	400 mg PO bid	2 years for the time of report	Stable disease with time-to-progression 5.7 month; decrease of tumor marker CA 19-9; decrease of bilirubin level and increase of liver synthesis parameters	Mild diarrhea, fatigue and skin toxicity; no dose reduction or interruption were made	[12]
Sorafenib	1	400 mg PO bid; 7 days cessation after 1 year and dose reduction to 200 mg	4 years for the time of report	Stable disease; decrease of bilirubin level	Mild diarrhea, desquamation rush. Grade 1 hand-foot syndrome, mild thrombocytopenia (required 7 days cessation)	[11]
Sorafenib	44	400 mg PO bid	1.8-month median duration	Median time to progression 5.6 month; median overall survival 5.7 month; disease control rate at 3 months 15.9%	Mild diarrhea, fatigue, hand-foot syndrome	[14]
Sorafenib	15	400 mg PO bid	3.2-month median duration	Median time to progression 3.2 month; median overall survival 5.7 month; disease control rate at 3 months 73.3%	Skin rush in 5 patients grade 3 hand-foot syndrome in 1 patient	[15]
Sorafenib	31	400 mg PO bid	2-month median duration	Disease control rate detected according suggested scheme 0%; median overall survival 9 month; median progression free survival 3 month	Grades 3 and 4 toxicities in 20 patients included: thrombosis/embolism, hypertension, fatigue, bilirubin evaluation, hand-foot syndrome	[16]
Sorafenib	46	400 mg PO bid	From 1 to 12 months	Disease control rate at 3 months 32.6%; median overall survival 4.4 month; median progression free survival 2.3 month	Hand-foot syndrome, skin rush, diarrhea, fatigue and thrombocytopenia	[17]
Pazopanib Trametinib	25	Pazopanib 800 mg qd Trametinib 2 mg qd	3-month median duration	Median overall survival 6.4 month; disease control rate detected according suggested scheme 75%; median progression free survival 3.6 month	Hypertension, fatigue, rash, diarrhea, nausea/vomiting, thrombocytopenia	[18]

In this report we describe a case of advanced metastatic CCA subsequently treated with TKI agents Sorafenib and Pazopanib, which resulted in 2-year survival period after initial diagnosis. Inhibitors were selected based on high throughput gene expression and molecular pathway activation analysis of the patient’s tumor biopsy, thus providing a personalized approach.

## Case

**Fig. 1:**
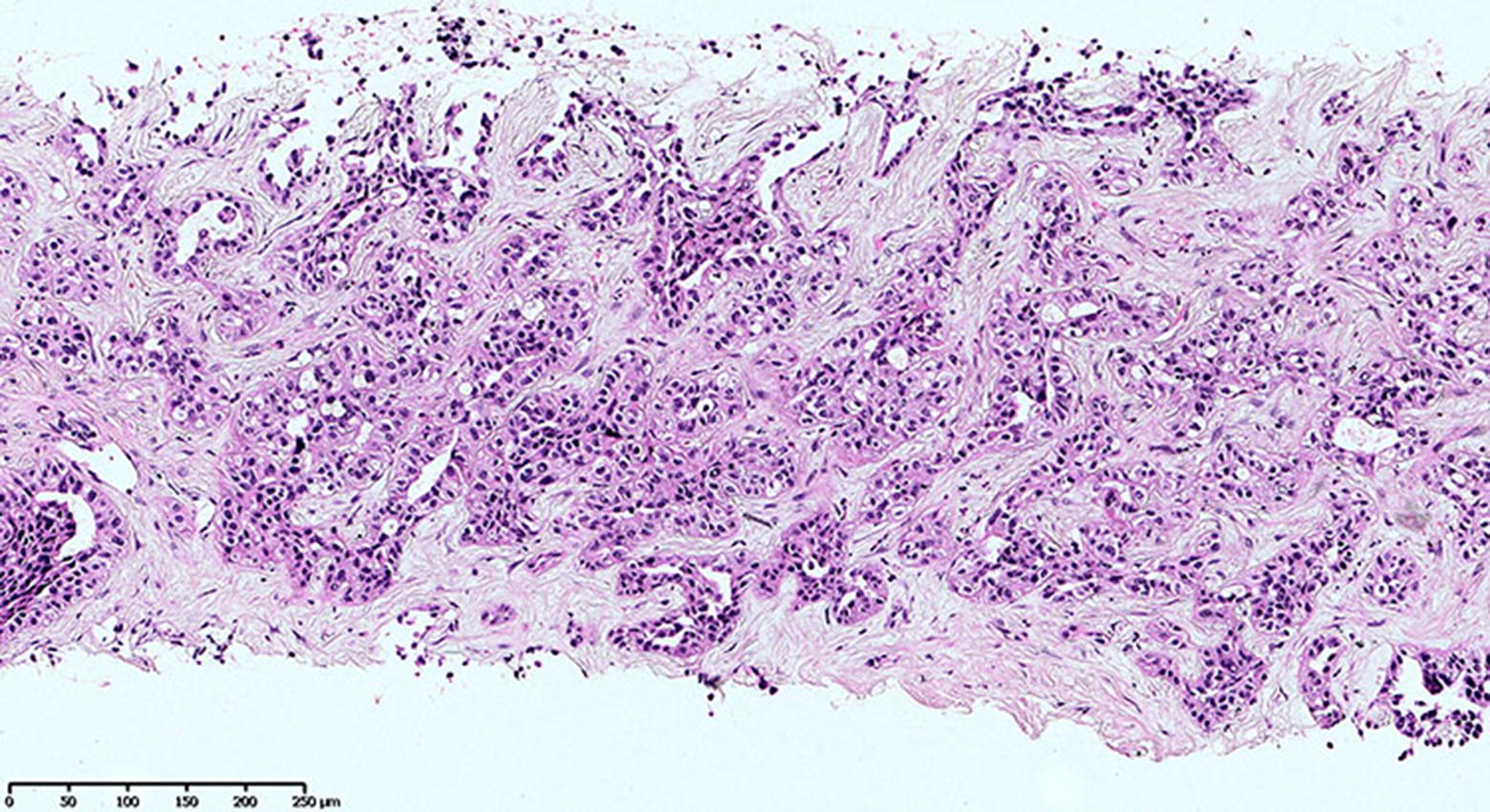
Hematoxylin and eosin (H&E) staining shows moderately differentiated intrahepatic cholangiocarcinoma (magnification ~ ×200)

**Fig. 2:**
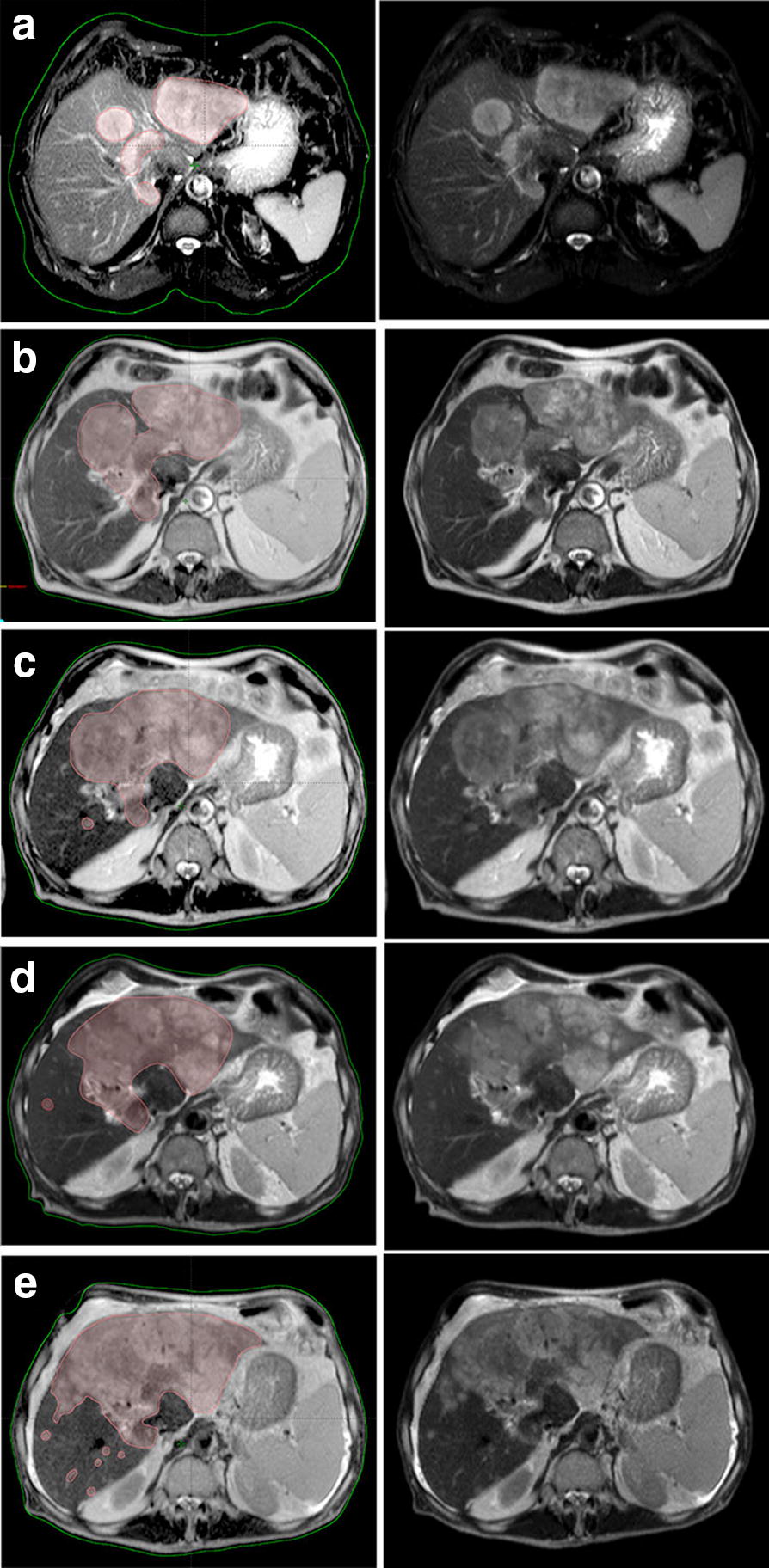
MRI tumor evaluation during treatment. Tumor area is highlighted on the left panel. Raw images are provided on the right panel. **a** Tumor evaluation at the stage of the initial CCA diagnosis (October 2015, was done with liver vein contrasting (7.5 ml Gadovist). Sum of diameters of target lesions equals 221 mm; **b** tumor progression after the best clinical practice care (July 2016). Sum of diameters of target lesions equals 278 mm (26% increase); **c** tumor growth during Sorafenib treatment (October 2016). Sum of diameters of target lesions equals 314 mm (16% increase); **d** tumor growth after Sorafenib treatment prior to start of Pazopanib (January 2017). Sum of diameters of target lesions equals 392 mm (35.3% increase). **e** Tumor progression after Pazopanib treatment (July 2017). Sum of diameters of target lesions equals 471 mm (35.7% increase if counting **a** as a reference or 20% increase if counting **d** as a reference)

The patient is a 72-year-old man with histologically confirmed moderately differentiated intrahepatic cholangiocarcinoma (Fig. 1). He was diagnosed in October 2015 with the following symptoms: moderate weight loss, pain in the right hypochondrium, loss of appetite and asthenia, with a Karnofsky scale index of 70%. MRI image at the time of diagnosis is shown on Fig. 2a. The tumor was not surgically removed because of advanced stage, multiple intrahepatic nodules and lung metastases.

**Fig. 3:**
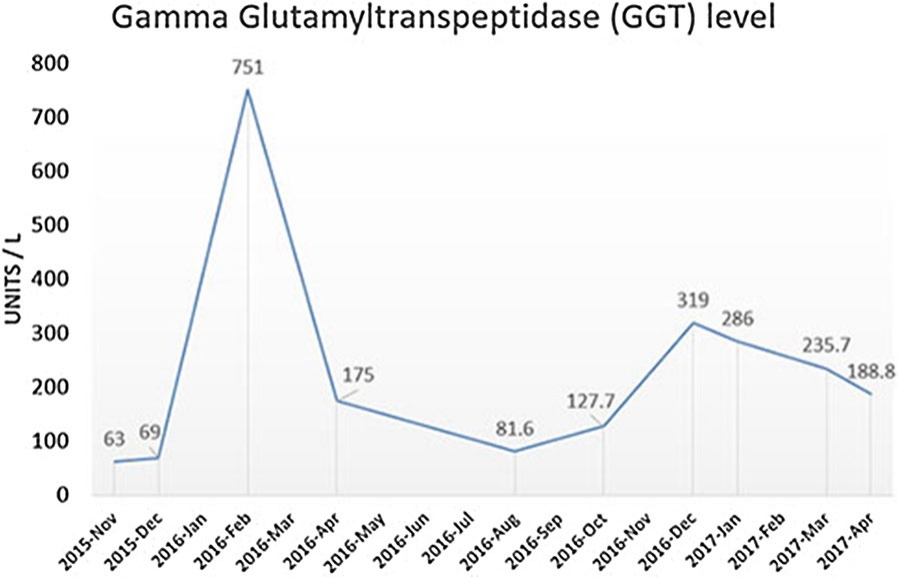
Serum gamma glutamyltranspeptidase levels during treatment

Four courses of chemotherapy (2 courses Gemcitabine in combination with Capecitabine and subsequent 2 courses Gemcitabine in combination with Cisplatin) were administered till May 2016. The treatment was poorly effective, and the tumor increased in size according to MRI (Fig. 2b); additional metastatic nodules appeared in the left and the right lobes with the spread to the bile duct, holedoch and into the gallbladder. Serum gamma glutamyltranspeptidase (GGT) level, which is associated with poor prognosis and tumor aggressiveness [21, 22], was significantly increased, when compared to pre-treatment levels (Fig. 3). Karnofsky scale index decreased to 60%. As the patient did not respond to the best clinical practice treatment, we decided to switch the medication and considered TKI inhibitors as further treatment option. Taking into account available data on differential response of CCA patients to TKIs we performed advanced molecular analysis of the tumor to support our choice and identify the most effective drug.

We profiled gene expression in formalin-fixed, paraffin-embedded (FFPE) patient’s tumor biopsy sample, obtained at the time of the first CCA diagnosis. Briefly total RNA was extracted using Ambion’s RecoverAll™ Total Nucleic Acid Isolation. Complete Whole Transcriptome Amplification WTA2 Kit (Sigma) was used for reverse transcription and library amplification. Hybridization was performed according to CustomArray ElectraSense™ Hybridization and Detection protocol. Hybridization efficiency was detected electrochemically using CustomArray ElectraSense™ Detection Kit and ElectraSense™ 4X2K/12K Reader.

**Fig. 4:**
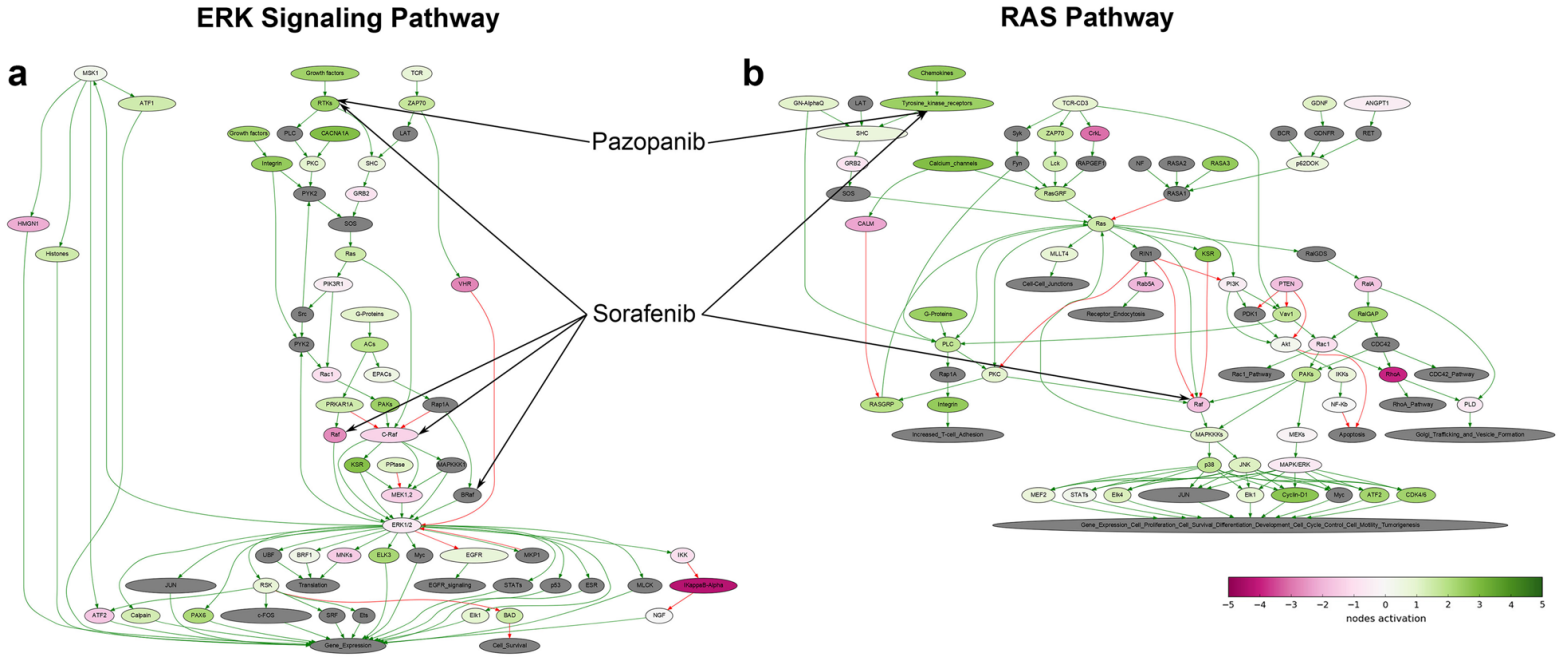
ERK and Ras signaling pathways were hyperactivated in the biopsy CCA tissue. Visualization was provided by Oncobox software. The pathways are shown as an interacting network, where green arrows indicate activation, red arrows—inhibition. Color depth of each node of the network corresponds to the logarithms of the case-to-normal (CNR) expression rate for each node, where “normal” is a geometric average between normal tissue samples, the scale represents extent of up/downregulation. The molecular targets of Sorafenib and Pazopanib are shown by black arrows

We next used bioinformatical software Oncobox to analyze gene expression data and to identify molecular pathways differentially regulated in the patient’s tumor sample [23]. Based on the abundance of gene transcripts for the molecular targets of anticancer drugs, Oncobox also makes it possible to generate a rating of target drugs potentially effective for the individual patients [24, 25]. Particularly, this analysis revealed that the ERK and Ras molecular signaling pathways were highly activated in the CCA patient’s tumor biopsy (Fig. 4), the predicted rating of the most effective target drugs is shown in Table 2. Regorafenib, a multi-tyrosine kinase inhibitor was on the top position of the rating. However, there were no published studies of Regorafenib efficacy and tolerability in CCA. At the same time, several case reports demonstrated efficiency of TKI target drug Sorafenib for CCA treatment [11–13]. We, therefore, decided to use Sorafenib as the next line therapy and it was prescribed to the patient (800 mg daily) in May 2016. Treatment with Sorafenib coincided with the decrease of serum GGT level. MRI analysis in October 2016 revealed moderate tumor growth, corresponding to disease stabilization (Fig. 2c). However, additional nodules occurred slightly below the xiphoid process in the diaphragm area. Therefore, disease progressed according to RECIST criteria. And, importantly, after Sorafenib treatment, the patient did not complain of pain in the right hypochondrium. Before Sorafenib treatment the patient received Tramadol (100 mg im once a day) and Fentanyl (75 µg/h, Duragesic transdermal tape). After 1 month of treatment with Sorafenib the pain medication was switched to Ketoral (30 mg im twice a day). Considering all the above-mentioned facts it was decided to continue Sorafenib treatment. MRI performed in January 2017 revealed progression of tumor growth and additional nodule in the left lung (Fig. 2d). In addition, the following side effects occurred: redness, swelling, pain on the palms of the hands and soles of the feet. GGT level increased up to 319 U/L in December 2016.

**Table 2:** Rating of target drugs provided by Oncobox test

Position	Drug
1	Regorafenib
2	*Sorafenib*
3	Sunitinib
4	*Pazopanib*
5	Axitinib
6	Vandetanib
7	Cabozantinib
8	Imatinib
9	Ziv-aflibercept
10	Dasatinib

The treatment regimen was next changed to Pazopanib, another TKI drug recommended based on the Oncobox rating. Sunitinib was not chosen because we attempted to eliminate the hand-foot syndrome, which occurred during Sorafenib administration. In the previous studies, Sunitinib treatment of CCA patients induced hand-foot syndrome in 43% of patients [26]. On the other hand, recent clinical trial of Pazopanib in combination with Trametinib in CCA did not report hand-foot syndrome as a side effect [27]. Pazopanib administration (800 mg daily) started since January 2017. The control MRI in July 2017 revealed progression in the lung nodes and 20% increase in sum of diameters of target lesions, which is a borderline between stabilization and progression according to RECIST (Fig. 2e). However, the change of treatment regimen resulted in elimination of Sorafenib side effects and general improvement of life quality. In addition, start of Pazopanib treatment coincided with a start of a trend towards decrease of serum GGT level (Fig. 3). As for October 2017 (2 years after initial diagnosis), the patient was alive and physically active, with Karnofsky scale 80%. Our patient passed away due to the liver failure in November 2017.

## Discussion

Our case report describes sequential use of TKI inhibitors Sorafenib and Pazopanib, which were selected based on personalized approach, for treating advanced CCA in patient who did not respond to standard therapy. Selected treatment improved patient life quality and survival period even though did not result in a response according to RECIST classification.

Available data on TKI usage in CCA patients underline that despite the potential benefit of such treatment not all patients respond equally. Therefore, it remains a clinical challenge to promptly identify potential responders. Selection of CCA patients, who may benefit from TKI treatment may be based on the molecular characteristics of tumor specimens.

Therefore, to support the usage of TKI in the current clinical case and select the most effective inhibitor we performed a molecular profile of patient tumor biopsy. Total RNA was extracted from FFPE tissue sample, obtained at the stage of the diagnosis, and gene expression was measured using microarray hybridization. Gene expression data was next used for calculating pathway activation scores and for building rating of target drugs using bioinformatical software Oncobox.

Performed analysis demonstrated that Ras and ERK signaling pathways were highly activated in the patient’s pathological tissue (Additional file 1: Table S1). These molecular pathways are implicated in various process linked with tumorigenesis such as cell proliferation, differentiation, survival and apoptosis [28]. Ras pathway activates ERK pathway, but also is tightly connected with stress response, cell motility and cytoskeleton rearrangements [29]. Normally both, ERK and Ras pathways are activated by binding of extracellular growth factors (like EGFR) to their receptor tyrosine kinases (RTKs). TKIs are capable of targeting RTKs, thus inhibiting cell proliferation and survival [30, 31].

Based on the observed molecular phenotype several TKIs were predicted to be effective in the current clinical case (Table 2). Considering both results of the bioinformatical analysis and available literature data on TKI efficiency in CCA, Sorafenib and then Pazopanib were prescribed to our patient. These drugs overlap in blocking FLT1, FLT4, KDR, C-Kit proto-oncogene and platelet derived growth factor receptor beta (PDGFRB). In addition, Pazopanib targets platelet derived growth factor receptor alpha (PDGFRA), while Sorafenib—B-Raf, C-Raf and ret proto-oncogenes, fibroblast growth factor receptor 1 (FGFR1) and FLT3. Both Pazopanib and Sorafenib are approved for treating advanced renal cell carcinoma (RCC); in addition, Sorafenib is approved for treatment of patients with unresectable hepatocellular carcinoma (HCC).

Thus, the performed analysis supported choice of treatment and predicted high chances of TKI efficiency for our patient. Even though the best clinical outcome across this case was progressive disease (according to RECIST), TKIs were beneficial for palliative care of metastatic CCA patient due to improvement of clinical parameters such as quality of life and survival. In addition, start of treatment with TKIs coincided with a trend towards decrease of serum GGT level.

The success of the most clinical trials of TKI in CCA was evaluated based on the time to progression or progression free survival and, therefore, disease progression was considered as an unsuccessful outcome and resulted in termination of treatment. In this case we report life quality improvement and prolonged survival with TKI therapy on the background of disease progression. This may point to the modest antitumor activity of TKIs in some patients, as also suggested by El-Khoueiry et al. [16]. Such activity, even if not capable to trigger the response, could potentially be sufficient for slowing down disease progression and, thus, life quality improvement.

## Additional file

**Additional file 1: Table S1.** Additional table.
